# Social Axioms and Individual Values as Predictors of COVID-19 Fear among University Students from Countries with Different Government Strategies for Managing the Pandemic

**DOI:** 10.11621/pir.2023.0103

**Published:** 2023-03-15

**Authors:** Nadezhda V. Murashcenkova

**Affiliations:** a HSE University, Moscow, Russia

**Keywords:** Social axioms, individual values, fear of COVID-19, government strategies for managing the pandemic, students

## Abstract

**Background:**

Effective prevention of psychological trauma by fear of COVID-19 requires the study of the relationships between the psychological and contextual factors that can influence the level of this fear. The social axioms, individual values, and government strategies for managing the pandemic have not yet been studied as a system of psychological and contextual factors contributing to COVID-19 fear.

**Objective:**

The aim of this study was to assess the level of COVID-19 fear and the characteristics of the relationships between the social axioms, individual values, and fear of COVID-19 among university students from countries with different government strategies for managing the pandemic.

**Design:**

University students from countries with different government strategies for managing the pandemic (208 Belarusians, 200 Kazakhstanis, and 250 Russians ages 18 to 25) participated in an anonymous online survey. The respondents filled in questionnaires that assessed their manifestations of COVID-19 fear (COVID-19 Fear Scale: FCV-19S) as the dependent variable; the “Social Axiom Questionnaire” (QSA-31) and the “Portrait Value Questionnaire” (ESS-21) measured the social axioms and individual values as the independent variables.

**Results:**

Fear of COVID-19 reached a higher level among the students from the countries with the weakest (Belarus) and the strongest (Kazakhstan) restrictive measures during the pandemic. Dysfunctional fear of COVID-19 was manifest among those Belarusian students who attached the greatest importance to self-enhancement values and the fate control axiom, and the least importance to the social complexity axiom, as well as among those Russian students for whom the religiosity social axiom was significant and the social complexity axiom was not. For Kazakhstani students, social axioms and values were not predictors of dysfunctional fear of COVID-19.

**Conclusion:**

The greatest contribution of social axioms and individual values to the experience of COVID-19 fear among the students was observed under conditions where the actions of the authorities were incompatible with the existing pandemic risks (in Belarus), as well as under conditions where a variable assessment of threat level was possible (in Russia).

## Introduction

The COVID-19 pandemic has given scientists the important task of determining the factors that can preserve the psychological well-being of populations in different countries under the threat of the disease (Bavel et al., 2020). One of the ways of solving this task is to identify the contextual and psychological factors that increase fear of COVID-19 among students, who are a high-risk group for the adverse effects of the pandemic (Deng et al., 2021). Students faced significant lifestyle changes during the pandemic (Aristovnik et al., 2020). These included adaptation to online learning (Pokhrel & Chhetri, 2021), changes in academic habits, social exclusion, and limited contact with peers (Aristovnik et al., 2020). A meta-analysis of 89 contemporary studies (Deng et al., 2021) confirmed an increase in anxiety and depression symptoms and sleep disorders among university students in different countries during the pandemic. Another meta-analysis focused on assessing the aggregate mean of coronavirus infection fear among college students of different countries (Wang et al., 2022). The study confirmed the importance of developing and implementing preventive mental health programs for college students during and after the pandemic.

The COVID-19 pandemic was a very strong stressor associated with the risk of causing the occurrence and long-term presence of different mental health disorders in the population (Cohen-Louck & Levy, 2021). Fear of COVID-19 was the core psychological novelty brought by the pandemic (Mertens et al., 2021), which increased the risk of psychological trauma for the population. Fear is a person’s adaptive response to danger. Fear of COVID-19 may lead people to behave cautiously during the pandemic (Harper et al., 2021; Pakpour & Griffiths, 2020). However, a prolonged experience and/or high level of COVID-19 fear can have an extremely damaging effect (Asmundson & Taylor, 2020; Ornell et al., 2020; Ren et al., 2020; Satici et al., 2020; Schimmenti et al., 2020). At the individual level, it can manifest itself in the development of anxiety-related disorders, depression, suicidal thoughts, and post-traumatic stress (Asmundson & Taylor, 2020; Ornell et al., 2020; Satici et al., 2020). At the social level, panic and xenophobia may spread (Ren et al., 2020; Schimmenti et al., 2020). Fear of COVID-19 is particularly dangerous because it can increase the damage from the disease itself (Ren et al., 2020).

Fear of COVID-19 is a complex multidimensional construct that includes various components (Mertens et al., 2021) and is measured by different psychological questionnaires (Ahorsu et al., 2022; Arpaci et al., 2020; Mertens et al., 2020; Schimmenti et al., 2020; Taylor et al., 2020). In our study, we researched fear about one’s own health and fear of getting infected with the coronavirus, namely the psychophysiological and psycho-emotional manifestations of COVID-19 fear. The assessment of psychophysiological and psycho-emotional manifestations of fear is important for differentiating functional and dysfunctional fears of COVID-19 (Harper, 2021; Solymosi et al., 2021). The markers of dysfunctional fear are primarily its psychophysiological manifestations (Hyde et al., 2019). Therefore, the ability to predict traumatization by fear of COVID-19 implies that we first analyze the level of its psychophysiological manifestations and the predictors of these manifestations.

We understand fear as an emotion based on experience and cognitive processing (Barrett, 2017) related to the assessment and interpretation of events (Lazarus, 1996). These assessments and interpretations depend on both external context and internal psychological factors (Lazarus & Folkman, 1984). The strategies governments adopted for managing the pandemic situation may have contributed to the development of COVID-19 fear, acting as significant external contextual factors (Al-Mahadin, 2020; Odintsova et al., 2021). In a COVID-19 threat situation, different countries responded in different ways and implemented different pandemic management strategies: these included the state-level strategy of pandemic denial, as in Belarus (Karáth, 2020; Odintsova et al., 2021; Shpakou et al., 2021); the introduction of a self-isolation regime, as in Russia (Reshetnikov et al., 2020); and the declaration of a state of emergency, as in Kazakhstan (Abramov et al., 2022). Meanwhile, the key parameter differentiating government strategies of managing the pandemic, which were significant in terms of their impact on the psychological state of citizens, was the degree of severity of the restrictive measures imposed on the population (Odintsova et al., 2021; Hale et al., 2020).

Internal factors can include social axioms and individual values, which act as filters of threat perception (Leung & Bond, 2009; Schwartz, 2015). Psychological research shows that social axioms and values are powerful psychological factors that influence people’s attitudes and behaviors in various spheres of activity (Leung & Bond, 2009; Schwartz, 2015), including disease risk assessment and the formation of different fears (Boehnke & Schwartz, 1997; Frink et al., 2004; Hui et al., 2007; Li et al., 2021; Schwartz et al., 2000; Tong et al., 2020). However, as far as we know, no published works have assessed the contribution of social axioms and individual values to fear of COVID-19 among students from countries with different government strategies for managing the pandemic.

Social axioms and individual values represent two distinct but interrelated types of psychological constructs (Leung et al., 2007). Both have the function of choosing and regulating people’s attitudes and behavior in different situations (Leung & Bond, 2009; Schwartz, 2015). People’s beliefs about the social world complement their motives for achieving various goals (Bond et al., 2004a). In this regard, the comprehensive study of social axioms and individual values can contribute to a better understanding of the mechanisms of people’s attitudes and behavior in complex situations requiring problem solving and adaptation (Bond et al., 2004a).

According to the theory developed by Michael Bond and Kwok Leung, social axioms are generalized beliefs about oneself, the social and physical environment, or the spiritual world, and are in the form of an assertion about the relationship between two entities or concepts (Leung et al., 2002). These beliefs are universal and determine the behavior and attitudes of people in different situations (Leung & Bond, 2009). Social axioms’ functions relate to people’s ability to adapt and survive (Bond et al., 2004b).

There are studies on the functioning of social axioms during the COVID-19 pandemic. For example, a Chinese sample (18-85 years old) showed a positive association of the fate control axiom with a high perception of the risk of coronavirus disease (Li et al., 2021). On a sample of Russians between 17 and 80 years of age, a positive correlation was found between a belief in conspiracy theories about COVID-19’s origin and the social cynicism axiom (Nestik et al., 2020). In another study on a Chinese sample (18 to 87 years old), a negative link between taking precautions in relation to COVID-19 and the social cynicism axiom was found (Tong et al., 2020). In this sample, the positive link between the reward for application axiom and taking precautions in relation to COVID-19 was also discovered (Tong et al., 2020). Therefore, the existing research confirms the significant role of social axioms in shaping people’s attitudes toward the pandemic and people’s behavior during this period.

Basic individual values, according to the theory by Shalom Schwartz, are motivational trans-situational goals that are the directing principles in people’s lives and influence their ideas, attitudes, and behavior (Schwartz, 1992; Schwartz et al., 2012). Schwartz considers values as beliefs inseparably related to affect (2015). Considering the theoretical aspects of the relationship between values and worries, Schwartz and colleagues (2000) underline that the same situation may provoke very different interpretations from people with varying value priorities. Values priorities impact worries, focusing individual attention and perception toward situations that threaten these values. A person’s perception of a threat to the realization of values important to them tends to elicit negative affective reactions (Schwartz, 2015). In the motivational approach to emotions, there is a similar idea, according to which fear arises when a person is prevented from achieving his/her desired goals (Lazarus, 1991). At the same time, Schwartz (2014) emphasizes that people for whom conservation values are more important than the values of openness to change can be physiologically more sensitive to negative and/or exciting environmental features. Thus, we can conclude that the link between values and fear can be mediated by two parameters, *i.e*., the significance of the values for the person and their content.

According to the basic provisions of Schwartz’s theory, conservation and self-enhancement values are generally related to avoiding or controlling anxiety (Schwartz, 2015). The values of self-enhancement have significant links with micro worries (fears for oneself and loved ones) (Boehnke et al., 1998; Schwartz et al., 2000). There is scientific evidence (Daniel et al., 2022) that concerns about infection by the COVID-19 virus are related to the diminished importance of the openness to change values and the increasing importance of conservation values (data from the Australian adult sample). Among Brazilian respondents (mean age 38), researchers have found a link between worries about coronavirus infection and the security value (Fischer et al., 2021).

However, under the unique conditions of the pandemic, we cannot predict exactly how individual values and expressions of COVID-19 fear can be linked with different social contexts, namely, in countries with different strategies of managing the pandemic. At the same time, the study of the relationship between social axioms, values, and real fear of COVID-19 (rather than abstract fear) in a real pandemic (rather than in hypothetical conditions) in different social contexts is of particular interest. The forced closure of international borders during the pandemic created the conditions for the study of various psychological phenomena in physically isolated environments with different contextual factors. Under these circumstances, the relationships of social axioms, values, and fear of COVID-19 can have their own specificity. This can be a consequence of the specific interaction between personal characteristics and social context (Fischer et al., 2021).

Consequently, the objective of this study was to assess the level of COVID-19 fear and the characteristics of the relationships between the social axioms, individual values, and fear of COVID-19 among university students from countries with different government strategies of managing the pandemic. Due to the novelty and particularity of the problem, and the absence of previous research, our analysis is somewhat speculative (Swedberg, 2021) and based on empirical scientific results (data-driven approach) (Jack et al., 2018). We have not proposed special research hypotheses, but rather two research questions:

1. Do university students from countries with different government strategies of managing the pandemic differ in their psychophysiological and psycho-emotional manifestations of COVID-19 fear?
2. Do university students from countries with different government strategies of managing the pandemic differ in the relationship between COVID-19 fear and their social axioms and individual values?

## Methods

### Participants

We tried to minimize the possible influence of sociocultural factors on the characteristics of relationships of dependent and independent variables in the study. Therefore, we included Russian-speaking students who are citizens of post-Soviet countries into the sample. The participants in the study were university students of ages 18-25 from Belarus, Kazakhstan, and Russia. After the dissolution of the USSR, Belarus, Kazakhstan, and Russia have maintained close socio-economic and cultural ties. They are all member states of the Common Economic Space and the Customs Union. Both in Belarus and Kazakhstan, free legal use of the Russian language is governed by the state. In Belarus, the Russian language has the status of a second state language, while in Kazakhstan it has the status of an official language and is used by the authorities and local governments on an equal footing with the State language (Kazakh).

We used a cross-sectional correlation design in the study. All respondents were Russian-speaking citizens and residents of their countries. The online link to the questionnaire was distributed to potential respondents by teachers and students from universities in the three countries. The total number of completed online questionnaires was 1,723. We removed the questionnaires that were partially filled out and did not match the sample parameters. After this, the basic sample included 987 students (208 Belarusians, 200 Kazakhstanis, and 579 Russians). For this study, which involved multi-group analysis, we reduced the sample of Russians to 250. Using stratified selection, the sample of Russian students was balanced by basic sociodemographic parameters with the samples of Belarusian and Kazakhstani students. Accordingly, the sample of this study included 208 Belarusians, 200 Kazakhstanis, and 250 Russians.

*Table 1* presents the age and gender composition of the three samples, as well as the cities of residence and the personal experience of the respondents with the coronavirus.

**Table 1:** Sample Composition

Citizenship (place of residence)	N	Age		Males (%)	Personal experience with the coronavirus
		*M*	*SD*		(I was sick myself) (%)
Belarusians (Minsk, Grodno, Vitebsk)	208	19.8	1.9	25.0	28.8
Kazakhstanis (Nur-Sultan, Pavlodar, Ust-Kamenogorsk)	200	20.5	1.9	26.0	11.0
Russians (Moscow, Saint Petersburg, Khabarovsk, Omsk, Penza, Smolensk)	250	20.0	1.5	25.2	29.2

Students majoring in humanities, engineering, and economics participated in the study (according to the samples: 87.5%, 8.2%, and 4.3% in Belarus; 68.5%, 22.5%, and 9.0% in Kazakhstan; and 70.4%, 8.0%, and 21.6% in Russia, respectively). Almost half of the respondents in each country answered that they did not belong to any religious denomination (45.6% Russians, 41.5% Kazakhstanis, and 49.0% Belarusians). Orthodox Christianity was the dominant religion in the Russian (46.4%) and Belarusian (40.9%) samples, while in the Kazakhstani sample it was Islam (39.5%).

### Procedure

Empirical data were collected from January 2021 to April 2021 in an anonymous survey on the anketolog.ru platform. Before completing the questionnaire, the respondents gave informed consent to participate in the study. The respondents volunteered to participate in the study and did not receive a reward.

According to the weekly epidemiological reports of the World Health Organization, during the time of data collection, the number of people infected and deceased from the coronavirus increased in all three countries (Weekly epidemiological update — 27 January 2021; Weekly epidemiological update on COVID-19 — 20 April 2021). In the pre-data-collection phase (2020), Belarus demonstrated a state-level strategy of pandemic denial (Karáth, 2020; Odintsova et al., 2021; Shpakou et al., 2021). Kazakhstan was the first among the three states to introduce restrictive measures and the only country to introduce a state of emergency (Abramov et al., 2022). A self-isolation regime was declared in Russia (Reshetnikov et al., 2020). At the time of the online survey, the three countries differed in their COVID-19 Stringency Index, which was extracted from the Oxford COVID-19 Government Response Tracker (Hale et al., 2020). At the time of the start of the online survey (January 2021), Belarus had the lowest level of restrictive measures (43), the highest level of restrictive measures was in Kazakhstan (69), and the intermediate level was observed in Russia (50). At the end of the empirical data collection (April 2021), the restrictive measures indicator remained the highest in Kazakhstan (63). In Belarus (42) and Russia (42), these parameters were equal.

### Measures

The online survey included validated and reliable psychological questionnaires. The dependent variable in our study was the fear of COVID-19, and the independent variables were social axioms and individual values. Additionally, the study collected the socio-demographic parameters of the respondents, such as gender, age, and economic status. Moreover, we assessed their level of religiosity and their personal experience with the pandemic. In the online questionnaire, the respondents also added information on citizenship, ethnic identity, place of residence, and the university major. We controlled the variables, such as gender, age, economic status, level of religiosity, and experience with the coronavirus that might have had a correlation with fear of COVID-19, social axioms, and individual values in our study.

#### Social Axioms

To assess social axioms, we used the “Social Axiom Questionnaire” (QSA-31), developed and tested by A.N. Tatarko and N.M. Lebedeva (Tatarko & Lebedeva, 2020). This is a shortened version of the full Russian-language version of Bond’s and Leung’s “Social axioms” questionnaire (Tatarko & Lebedeva, 2011). The five-factor structure of the social axioms model has been confirmed in this questionnaire. It has high reliability and coherence. According to the questionnaire keys (Tatarko & Lebedeva, 2020), data processing calculated the mean values for five social axioms: social cynicism; fate control; religiosity; reward for application; and social complexity. Cronbach’s alphas for these scales were: Belarusian students, α = 0.66/0.78/0.91/0.81/0.69; Kazakhstani students, α = 0.72/0.75/0.93/0.82/0.66; and Russian students, α = 0.64/0.73/0.91/0.80/0.58, respectively.

#### Individual Values

To assess individual values, we used the abridged version of the “Portrait Value Questionnaire” by Schwartz (ESS-21), developed for the European Social Survey (Schwartz et al., 2001). The questionnaire comprises 21 items and measures 10 basic values and four higher order values. In this study, the variables were higher order values. According to the recommendations by Schwartz (Schwartz, 2003), and in accordance with the objective and method (MGSEM) of the study, we calculated mean values for four higher order values. Cronbach’s alphas for the Conservation values/ Openness to change values scales were: Belarusians, α = 0.63/0.67; Kazakhstanis, α = 0.58/0.68; and Russians, α = 0.68/0.69. Cronbach’s alphas for the Self-Enhancement/ Self-Transcendence values scales were: Belarusians, α = 0.71/0.66; Kazakhstanis, α = 0.67/0.58; and Russians, α = 0.74/0.70.

#### Fear of COVID-19

To measure the expression of COVID-19 fear, we used the COVID-19 Fear Scale (FCV-19S) developed by a group of scientists from the United Kingdom, Hong Kong, Iran, and Sweden (Ahorsu et al., 2022). The Russian-language version of the questionnaire was tested in a Russian-language sample in Russia and Belarus (Reznik et al., 2021). The questionnaire includes seven items. The respondents indicated their level of agreement with the items using a 5-point scale: 1 = “strongly disagree,” 2 = “disagree,” 3 = “neither agree nor disagree,” 4 = “agree,” and 5 = “strongly agree.” The sum of the items was then calculated. The higher the score, the greater was the fear of COVID-19.

Some academic papers compare the one-factor (Al-Shannaq et al., 2021; Mailliez et al., 2021) and the two-factor (Chen et al., 2022; Tzur Bitan et al., 2020) structure of this questionnaire. The advantage of using the two-factor structure of the Fear of COVID-19 Scale is the possibility of differentiating the psychophysiological and psycho-emotional manifestations of COVID-19 fear in the assessment (Chen et al., 2022; Tzur Bitan et al., 2020). In our study, we use a two-factor structure. The first factor includes third, sixth, and seventh items of the COVID-19 Fear Scale and shows the psychophysiological manifestations of COVID-19 fear (*e.g.*, “My hands become clammy when I think about coronavirus-19”). The second factor contains the first, second, fourth, and fifth items of this questionnaire and shows the psycho-emotional manifestations of this fear (*e.g.*, “I am most afraid of coronavirus-19”).

In our Multigroup Structural Equation Modeling (MGSEM) study, we modulated the dependent variables (the psychophysiological and the psycho-emotional manifestations of COVID-19 fear) as two latent factors represented by three and four measured variables. In addition, we analyzed the reliability and coherence of these two scales. Cronbach’s alphas for scales of the psychophysiological manifestations of COVID-19 fear and psycho-emotional manifestations of COVID-19 fear were: Belarusians, α = 0.88/0.80; Kazakhstanis, α = 0.80/0.79; and Russians, α = 0.80/0.75, respectively.

### Statistical Analysis

For statistical analysis we used SPSS Statistics version 23 and AMOS version 23. We calculated the psychometric measures of the scales (Cronbach’s alpha) and descriptive statistics for the dependent and independent variables. Additionally, we calculated the significance of the mean value differences (ANOVA with a post-hoc test) for each basic variable among the three samples. For testing the assumptions of Multi-Group Structural Equation Modeling (MGSEM), AMOS version 23 was used.

## Results

In the first step of data processing, we calculated the internal consistency of the scales (Cronbach’s alpha). All scales had sufficient internal consistency in each of the three samples (Nasledov, 2013). In the second step, we calculated the descriptive statistics and the significance of mean value differences for each basic variable across the three samples. *Table 2* shows the descriptive statistics and the significance of mean value differences (ANOVA with post-hoc test) of basic variables in the three samples.

**Table 2:** Means, Standard Deviations, Differences (Belarusians, Kazakhstanis, and Russians)

	Belarusians	Kazakhstanis	Russians
Variables	M(SD)	M(SD)	M(SD)
1. Reward for application	4.12(0.62)k^***^	4.44(0.55)r^***^,b^***^	4.20(0.57)k^***^
2. Social complexity	4.01(0.34)	4.02(0.38)	4.04(0.33)
3. Social cynicism	2.94(0.61)	2.94(0.69)	2.93(0.61)
4. Religiosity	2.67(0.96)k^*^	2.92(1.09)b^*^	2.72(0.98)
5. Fate control	2.34(0.81)k^**^	2.56(0.76)b^**^	2.41(0.73)
6. Self-Transcendence values	4.55(0.77)	4.74(0.69)	4.62(0.78)
7. Openness to change values	4.44(0.72)	4.54(0.74)	4.50(0.73)
8. Self-Enhancement values	4.22(0.87)	4.17(0.92) r^*^	4.29(0.94) k^*^
9. Conservation values	3.84(0.78)	3.96(0.79)	3.80(0.81)
10. Psychophysiological tations of COVID-19 manifes- fear	4.70(2.54)r^***^	4.89(2.47)r^***^	3.98(1.67)k^***^,b^***^
11. Psycho-tions of emotional COVID-19 manifesta- fear	10.35(4.11) r^**^	10.36(4.21)r^**^	9.22(3.76)k^**^, b^**^

*Note. b = The statistically significant difference with Belarusians. k = The statistically significant difference with Kazakhstanis. r = The statistically significant difference with Russians.* ^*^p < .05; ^**^p < .01; ^***^p < .001.

We did not find any statistically significant differences in social axioms and individual values between Belarusian and Russian youth. However, Kazakhstani students showed more commitment to the reward for application axiom than their Belarusian and Russian counterparts. In addition, Kazakhstani students showed more commitment to social axioms, such as religiosity and fate control than their Belarusian colleagues. Moreover, there were some differences in individual values among the students from Kazakhstan and Russia. Kazakhstani students were more predisposed to self-enhancement values than Russian students. Despite the differences identified, the students from all three countries had the same hierarchical structure (according to means) of social axioms (in ascending order: fate control, religiosity, social cynicism, social complexity, reward for application) and of higher order values (in ascending order: conservation, self-enhancement, openness to change, and self-transcendence values).

We did not find any statistically significant differences in the manifestations of COVID-19 fear between Belarusian and Kazakhstani youth. However, Russian students showed a lower level of psychophysiological and psycho-emotional manifestations of COVID-19 fear than the Belarusian and Kazakhstani students.

**Figure 1.**
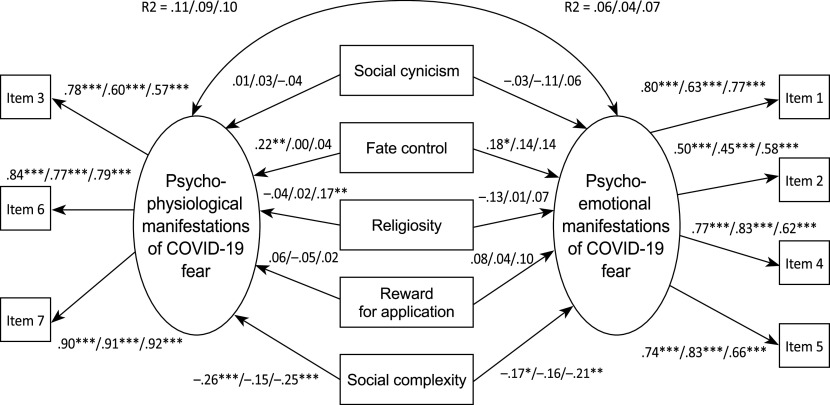
Standardized Coefficients (Unconstrained) for the Multi-Group Model (Controlling for Age, Gender, Economic Status, Level of Religiosity, and Personal Experience with COVID-19) of the Relationship Between Social Axioms and Fear of COVID-19 Across Students of Three Countries (Belarusians/Kazakhstanis/Russians)

Note. *p < .05; **p < .01; ***p < .001.

*Figure 1* presents a multi-group model of the relationship (unconstrained) of social axioms with the psychophysiological and the psycho-emotional manifestations of COVID-19 fear across the three countries. The sequence of coefficients is the following: Belarusians/Kazakhstanis/Russians.

According to the goodness-of-fit indices, this model fits the empirical data (see *Table 3*). There are configural, metric, and scalar invariance.

**Table 3:** Invariance for the Model of the Relationship Between Social Axioms and Fear of COVID-19 Across Students of Belarus, Kazakhstan, and Russia

Model	CFI	ΔCFI	RMSEA	PCLOSE	AIC	χ^2^	df	p
Unconstrained	0.955		0.035	1.000	517.881	205.881	114	< 0.001
Measurement weights	0.951	0.004	0.035	1.000	516.389	224.389	124	< 0.001
Measurement intercepts	0.958	0.007	0.031	1.000	488.389	224.389	138	< 0.001
Structural weights	0.959	0.004	0.029	1.000	467.484	243.484	158	< 0.001

*Note. CFI = comparative fit index; RMSEA = root mean square error of approximation; PCLOSE = p of Close Fit. AIC = Akaike information criterion; χ^2^ = chi-square; df = degrees of freedom; p = p-value.*

We discovered no statistically significant relationships between manifestations of COVID-19 fear and social axioms among Kazakhstani youth. Among Belarusian and Russian youth, social axioms contributed more to the explanation of psychophysiological manifestations of COVID-19 fear than psycho-emotional manifestations. Among Belarusian youth, the students who were committed to the axiom of fate control and did not share the belief in the social complexity of the world had larger psychophysiological manifestations of COVID-19 fear. The commitment to the axiom of fate control and low belief in social complexity were also predictors of psycho-emotional manifestations of COVID-19 fear among Belarusian students. However, the links of these predictors to psycho-emotional manifestations were weaker than with psychophysiological manifestations of this fear. The weak commitment to the social complexity axiom, combined with a strong belief in the beneficial influence of religion on society, were predictors of the psychophysiological manifestations of COVID-19 fear among Russian youth. Moreover, Russian students who did not believe in the complexity of the social world also had high psycho-emotional manifestations of this fear.

**Figure 2.**
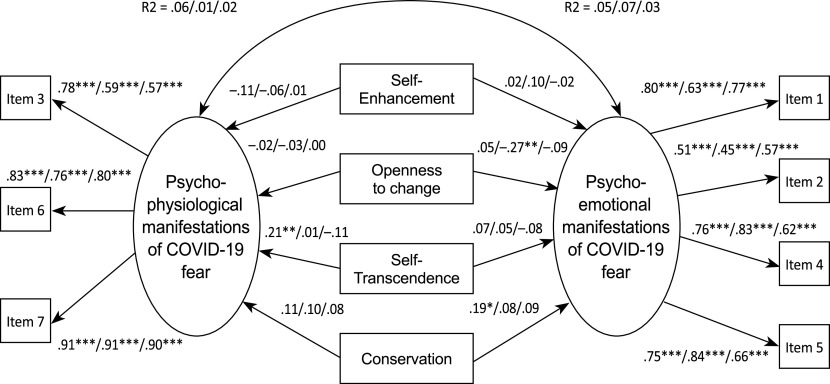
Standardized Coefficients (Unconstrained) for the Multi-Group Model (Controlling for Age, Gender, Economic Status, Level of Religiosity, and Personal Experience with COVID-19) of the Relationship Between Individual Values and Fear of COVID-19 Across Students of Three Countries (Belarusians/Kazakhstanis/Russians)

Note. *p < .05; **p < .01; ***p < .001.

*Figure 2* visually represents the multi-group model of the relationship of values with the psychophysiological and psycho-emotional manifestations of COVID-19 fear across students of the three countries. The sequence of coefficients is the following: Belarusians/Kazakhstanis/Russians.

As in the previous model (see *Table 3*), according to the goodness-of-fit indices, this model fit the empirical data (see *Table 4*). There are configural, metric, and scalar invariances.

**Table 4:** Invariance for the Model of the Relationship Between Individual Values and Fear of COVID-19 Across Students of Three Countries

Model	CFI	ΔCFI	RMSEA	PCLOSE	AIC	χ^2^	df	p
Unconstrained	0.958		0.036	0.998	448.610	196.610	105	< 0.001
Measurement weights	0.954	0.004	0.037	0.999	447.522	215.522	115	< 0.001
Measurement intercepts	0.960	0.006	0.032	1.000	419.522	215.522	129	< 0.001
Structural weights	0.954	0.004	0.032	1.000	415.977	243.977	145	< 0.001

*Note. CFI = comparative fit index; RMSEA = root mean square error of approximation; PCLOSE = p of Close Fit. AIC = Akaike information criterion; χ^2^ = chi-square; df = degrees of freedom; p = p-value.*

We discovered only one statistically significant relationship between the values and the psychophysiological manifestations of COVID-19 fear, and two statistically significant relationships between the values and the psycho-emotional manifestations of this fear. In the first case, there was a positive link between the psychophysiological manifestations of COVID-19 fear and self-enhancement values among Belarusian youth. In the second case, there was a negative relationship between the psycho-emotional manifestations and openness to change values among Kazakhstani youth, and the positive relationship between the psycho-emotional manifestations and conservation values among Belarusian youth.

Individual values (R^2^_be_=.06, R^2^_kz_=.01, R2_ru_=.02) contributed less to the explanation of psychophysiological manifestations of COVID-19 fear than social axioms (R^_2_^_be_=.11, R^2^_kz_=.09, R^2^_ru_=.10) in all three samples, respectively. In addition, they (R^2^_be_=.05, R^2^_ru_=.03) explained a smaller percentage of the variance of psycho-emotional manifestations of this fear than social axioms did (R^2^_be_=.06, R^2^_ru_=.07) in the Belarusian and Russian samples. Individual values (R^2^_kz_=.07) explained a larger percentage of the variance of psycho-emotional manifestations of this fear than social axioms (R^2^_kz_=.04) in the Kazakhstani sample only.

## Discussion

We found that fear of COVID-19 (both its psychophysiological and psycho-emotional manifestations) was more distinct among the students from the countries with the weakest (Belarus) and strongest (Kazakhstan) restrictive measures during the pandemic, which is consistent with existing studies (Al-Mahadin, 2020; Odintsova et al., 2021). In this regard, we can conclude that the risk of psychological trauma to youth caused by fear of COVID-19 was higher in Belarus and Kazakhstan than in Russia, *i.e.*, in states, which have implemented polar-opposite strategies of managing the pandemic. In Belarus, the strategy was the denial of the pandemic, and in Kazakhstan, it was the establishment of a state of emergency.

In Belarus, the mechanism of increased fear was most likely related to cognitive dissonance. It arose when information transmitted by the authorities did not cohere with information received by the people from other sources (Internet, social networks, acquaintances, etc.). This could contribute to increasing tension and anxiety about the insufficiency of measures, the level of protection and control of the situation by the State, and could provoke the actualization of fear. At the same time, it is worth noting that youth are the most Internet-oriented part of the population, with access to a wide variety of sources of information that they actively use. In the Kazakhstani case, we are rather dealing with a mechanism of escalating fear, as the measures applied by the State are associated with a high level of existing risks and threats (Han et al., 2021).

We discovered no common relationships between social axioms, individual values, and COVID-19 fear among Russian-speaking students in the post-Soviet countries with different strategies of managing the pandemic and the degree of restrictive measures severity. This leads us to the conclusion that government policies of containing the pandemic can mediate the links between fundamental psychological constructs and fear of coronavirus infection. Social axioms had the most influence on dysfunctional fear in students of Belarus and Russia, *i.e.,* countries with weak and moderate restrictive measures during the pandemic. Among Kazakhstani students, we did not discover any relations between social axioms and fear of COVID-19, although the level of dysfunctional fear among Kazakhstanis, similarly to Belarusians, was significantly higher than among Russians. It is likely that the state of emergency and state-imposed measures (as a contextual factor) in Kazakhstanis had a stronger impact on the growth of fear than social axioms. Perhaps, when the high threat of the pandemic is recognized at the state level, as was the case in Kazakhstan, and does not imply other interpretations of the situation, the diversity of psychological characteristics of the people with dysfunctional fear is broader. Therefore, we could not detect clear psychological profiles of the links between dysfunctional fear of coronavirus infection and specific social axioms. For Belarusian and Russian students, on the contrary, we identified such profiles and can explain them based on the results of already existing studies.

Among Belarusians, fear of coronavirus infection (both its psychophysiological and psycho-emotional manifestations) was positively associated with the fate control axiom and negatively associated with the social complexity axiom. An earlier study showed that social axioms can be protective mechanisms that defend people from fears (Hui et al., 2007). The social complexity axiom is a cognitive resource that is linked to coping strategies (Bond et al., 2004a), and is particularly relevant when adapting to new and unusual conditions such as the pandemic (Hui & Hui, 2009). In turn, the fate control axiom is connected with distancing from trying to solve problems and a distorted perception (wishful thinking) (Bond et al., 2004a). As the results of our research demonstrate, the belief in high control by fate, combined with a low belief in the complexity of the social world, can lead to a fatalistic assessment of the present, provoking an increase in fear of COVID-19 among Belarusian students. Let us recall that these links are manifested among the students of Belarus, a country that denied the danger of the pandemic at the state level and applied the weakest restrictive measures of the three countries that we studied.

Among Russian students, psycho-emotional manifestations of COVID-19 fear were negatively related with the social complexity axiom. At the same time, psychophysiological manifestations of COVID-19 fear among Russian youth had both a negative link with the social complexity axiom and a positive relationship with the religiosity axiom. That is, the dysfunctional fear of coronavirus infection (psychophysiological markers) had a higher level among those Russian students who were convinced of the beneficial influence of religion on society and underestimated the complexity of social peace and human behavior.

Let us consider a possible explanation for this. Young people with a positive attitude towards religion (and most likely with a religious world view) may view the pandemic not as a situation that occurs objectively due to the confluence of different circumstances, but as punishment, retribution, and chastisement from above. These perceptions can lead to increased anxiety and fear, as such ideas of the source of the threat are also linked to perceptions of its uncontrollability. At the same time, the connections found may indirectly indicate that Russian respondents with high levels of COVID-19 fear prefer a religious way of knowing and explaining the world to a scientific way of knowing, *i.e.*, faith versus verification and proof of assumptions. In this case, we can talk about the resource potential of the social complexity axiom, which is related to the scientific way of understanding the world and is associated with active coping in the prevention of psychological traumatization by fear among Russian and Belarusian students.

The positive link of the religiosity axiom with the fear of COVID-19, discovered only among Russian students, suggests that this relationship may be due to sociocultural factors. In this case, however, we tend to explain the link on the basis of the different government strategies for managing the pandemic in the three countries. The choice of this explanatory approach, which is based on the analysis of the social context rather than the sociocultural characteristics of the respondents, was prompted by the following analysis.

In the Kazakhstani sample, we found no relationship between the religiosity axiom and fear of COVID-19, despite the fact that this axiom (as well as the level of religiosity in general) was most strongly expressed in the Kazakhstani sample. Using the contextual approach, we explain the positive link between the religiosity axiom and fear of COVID-19 among Russian youth as manifesting an intolerance towards uncertainty and its relationship with attitude towards religion (Ulybina & Baklanova, 2019). Russia’s chosen strategy of moderating restrictive measures during the pandemic (compared to Kazakhstan, where the authorities introduced a state of emergency), increased the level of uncertainty, allowing the population to have different interpretations of the level of the current COVID-19 threat. In turn, research results confirm that loss of a sense of certainty can lead to increased religiosity (Laurin et al., 2008; Wichman, 2010). Therefore, probably the highest level of COVID-19 fear in the Russian sample was demonstrated by those respondents who had a low level of tolerance of uncertainty, and who referred to religion as a valuable system of understandable explanatory principles and meanings, which replaces the need for independent analysis of the situation.

It is known that religion provides people a worldview with elaborate information-processing schemes, offers normative practices of everyday life, and provides clear rules of behavior. In this way, religion helps people cope with uncertainty, creates a sense of order, and helps to reduce anxiety (Shaw et al., 2005). Moreover, scientific evidence has shown that religiosity and intolerance of uncertainty have common physiological grounds, and religious belief reduces brain reactions associated with anxiety (Inzlicht et al., 2009). At the same time, we do not rule out the possibility of the reverse influence, *i.e*., of fear as an independent variable being a predictor of axioms as dependent variables. Perhaps, among Russian students with a high level of dysfunctional fear and underestimation of the social world complexity, the recognition of the beneficial influence of religion on society was a consequence of the search for additional resources to control anxiety and cope with the pandemic.

In turn, the strategy for managing the pandemic in Kazakhstan, associated with severe restrictive measures, did not allow for variations in interpretations of the existing threat level. This is probably why the link between the religiosity axiom and the COVID-19 fear was not discovered in the Kazakhstani sample. In Belarus, we saw a different situation. The state-level strategy of pandemic denial, combined with the objective threat of COVID-19 in Belarus, could promote perceptions of the situation as particularly threatening and poorly controlled (especially among people with a low level of belief in the complexity of the world). These representations, in turn, may have led to an overestimation of risk, fatalism, and catastrophization among individual citizens, provoking the growth of dysfunctional COVID-19 fear. Perhaps, that is why we saw a positive link between the fate control axiom and the manifestations of COVID-19 fear in the Belarusian sample.

It is also interesting that we did not find significant links between individual values and dysfunctional fear of coronavirus infection (psychophysiological markers) among Russian and Kazakhstani youth. At the same time, Russian students did not show significant relationships between values and the psycho-emotional manifestations of this fear. Meanwhile, among Kazakhstani students, the high level of psycho-emotional manifestations of COVID-19 fear was correlated with the denial of the openness to change values, and the low level of this fear was related to a preference for these values, respectively.

This is quite logical and can be explained from the standpoint of Schwartz’s theory. Schwartz (2014) emphasizes that people for whom the values of openness to change are more important than conservation values can be physiologically less sensitive to negative and/or exciting environmental features. Intrinsically, the openness to change values focus on growth and development, while the values of conservation relate to protection against anxiety. The people who attach special importance to the openness to change values tend to embrace novelty, variation, and new impressions. Therefore, the pandemic could be perceived not as an alarming event by them, but as an opportunity to achieve meaningful goals.

However, here, we should pay attention to another aspect. By studying the real fear of coronavirus infection under the conditions of the pandemic among the respondents of the three countries, we found no relationship between values and dysfunctional fear across students from the two countries, namely Russia and Kazakhstan. This is inconsistent with the previous study, according to which micro and macro worries showed strong links to personal values (Schwartz et al., 2000). Probably, the relationships between fears and anxieties with values in hypothetical and real circumstances may differ, because of external contextual factors. At the same time, the links of values with COVID-19 fear found in the Belarusian sample were quite expected and can be explained by the basic provisions of Schwartz’s theory about the anxiety-avoidant values and the anxiety-free values. So, psychophysiological manifestations of COVID-19 fear among Belarusian students were positively associated with values of self-enhancement, and psycho-emotional manifestations were connected to conservation values. That is, fear was related to the values that are associated with self-protection, control, and avoiding anxiety.

Let us also emphasize that we do not rule out the possibility of a reverse influence of COVID-19 fear on individual values. This explanation is especially pertinent since some published studies have confirmed a change in people’s basic individual values under the influence of the pandemic (Daniel et al., 2021; Fischer et al., 2021). However, as part of this research, we are studying social axioms and individual values as predictors and information perception filters that can influence the formation of the fear of coronavirus infection.

Interestingly, individual values contributed less to the explanation of psychophysiological manifestations of COVID-19 fear than social axioms in all three samples. That is, the way a person perceives the world (social axioms) has a greater influence on the construction of fear in this case rather than what one aspires to (values). At the same time, psychological factors, both cultural (social axioms) and individual (values) levels, made the greatest contribution to the dysfunctional fear of COVID-19 among the students of Belarus. That is, this effect was observed among youth who were living under conditions of weak restrictive measures and denial of the pandemic by the state authorities, and a high risk of rapid and widespread infection.

According to the results of our research, social axioms and individual values play a significant role in the growth of COVID-19 fear when there is a clear mismatch between the official position of the state and the existing reality, *i.e*., weak restrictive measures in Belarus, as well as under the possibility of varied assessments of the level of current threats, and moderate restrictive measures in Russia. This can lead to increased uncertainty and actualize special psychological mechanisms of attribution and coping in people, triggering specific social axioms and individual values. We observed these effects in the Belarusian and Russian samples, but not in the Kazakhstani sample.

At the same time, the students from Belarus and Kazakhstan did not differ in the level of dysfunctional fear of COVID-19, and COVID-19 fear among students of these two countries was significantly higher than among Russian students. That is, both Kazakhstani and Belarusian students could be included in the risk group for psychological trauma by fear of COVID-19. However, as we see, the role of psychological factors in the development of COVID-19 fear differed among the students in these countries, which is important to take into account when organizing psychological assistance for the pandemic and the post-pandemic periods.

## Conclusion

This study enriches scientific knowledge in the field of assessing the influence of social axioms and individual values on the psychological well-being of people across different social contexts. The current study shows that the strategies for managing the pandemic can influence the level of COVID-19 fear and mediate links between this fear, social axioms, and individual values. Special attention should be paid to the population of countries and regions with the most severe and the least severe restrictive measures during the pandemic. As our research has shown, it is students from these types of countries who are at risk for psychological trauma by fear of COVID-19. In the context of future research, of particular interest are the psychological profiles of people who are especially afraid, or not at all afraid, of the COVID-19 in different sociocultural contexts, involving additional demographic, individual-psychological, and socio-psychological variables. In particular, considering the parameters of our study’s samples, estimating gender differences in the impact of independent variables on the level of COVID-19 fear is an important task for future research.

## Limitations

The present study had a number of limitations. We used cross-sectional correlation design, relied on self-report data, and used only quantitative data obtained predominantly with the help of the shortened versions of questionnaires. In addition, the limitations included the gender disparity within the three samples and convenience sampling. Due to the existing limitations and the fact that the study was exploratory, the conclusions of our research are probabilistic; and the results of the study are partially theoretical. We do not exclude the influence of other contextual factors on the discovered relationships. Additionally, we admit the possibility of another theoretical justification of the study, which may explain the inverse relationship between social axioms and values with fear of COVID-19.
